# ICTV Virus Taxonomy Profile: Simuloviridae 2023

**DOI:** 10.1099/jgv.0.001841

**Published:** 2023-04-24

**Authors:** Ying Liu, Shishen Du, Xiangdong Chen, Mike Dyall-Smith, Matti Jalasvuori, Hanna M. Oksanen, ICTV Report Consortium

**Affiliations:** 1 Archaeal Virology Unit, Institut Pasteur, Université Paris Cité, CNRS UMR6047, Paris, France; 2 Department of Microbiology, College of Life Sciences, Wuhan University, Wuhan 430072, PR China; 3 Computational Biology Group, Max Planck Institute of Biochemistry, Martinsried, Germany; 4 Veterinary Biosciences, Faculty of Veterinary and Agricultural Sciences, University of Melbourne, Parkville, Australia; 5 Department of Biological and Environmental Science, University of Jyväskylä, Jyväskylä, Finland; 6 Molecular and Integrative Biosciences Research Programme, Faculty of Biological and Environmental Sciences, University of Helsinki, Helsinki, Finland

**Keywords:** ICTV Report, taxonomy, *Simuloviridae*

## Abstract

The family *Simuloviridae* includes tailless icosahedral viruses with an internal lipid membrane. The capsid is constructed from two major capsid proteins, both with a single jelly-roll fold. The genome is a circular dsDNA molecule of 16–19 kb. All members infect halophilic archaea in the class Halobacteria (phylum Euryarchaeota) and are temperate viruses, their proviruses residing in host cells as extrachromosomal episomes. Once the lytic life cycle is triggered, production of virions causes cell lysis. This is a summary of the International Committee on Taxonomy of Viruses (ICTV) Report on the family *Simuloviridae,* which is available at ictv.global/report/simuloviridae.

## Virion

**Fig. 1.**
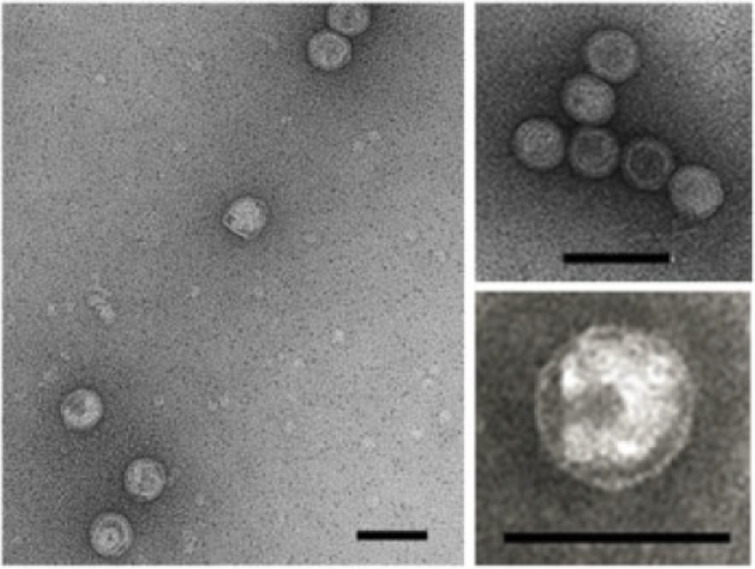
Electron micrographs of Saline Natrinema sp. J7-1 virus 1 virions. Bars, 100 nm. Reproduced with permission from Liu *et al*. [2].

Virions of Saline Natrinema sp. J7‐1 virus 1 are tailless icosahedra with a diameter of approximately 70 nm (Table 1, Fig. 1) constructed from two single jelly-roll major capsid proteins PB2 and PB6, and an internal membrane core enclosing the viral circular dsDNA genome [1,2].

**Table 1.** Characteristics of members of the family *Simuloviridae*

Example	Saline Natrinema sp. J7‐1 virus 1 (AY048850), species *Yingchengvirus SNJ1*, genus *Yingchengvirus*
Virion	Tailless icosahedral virion with an internal lipid membrane, diameter about 70 nm; two major capsid proteins, both with a single jelly-roll fold
Genome	Circular dsDNA of 16–19 kb
Replication	Rolling circle replication
Translation	Prokaryotic translation using viral mRNA and host ribosomes
Host range	Halophilic archaea of the genera *Natrinema* and *Haloterrigena*, order Natrialbales
Taxonomy	Realm *Varidnaviria*, kingdom *Helvetiavirae*, phylum *Dividoviricota*, class *Laserviricetes*, order *Halopanivirales*: one genus, three species

## Genome

**Fig. 2.**
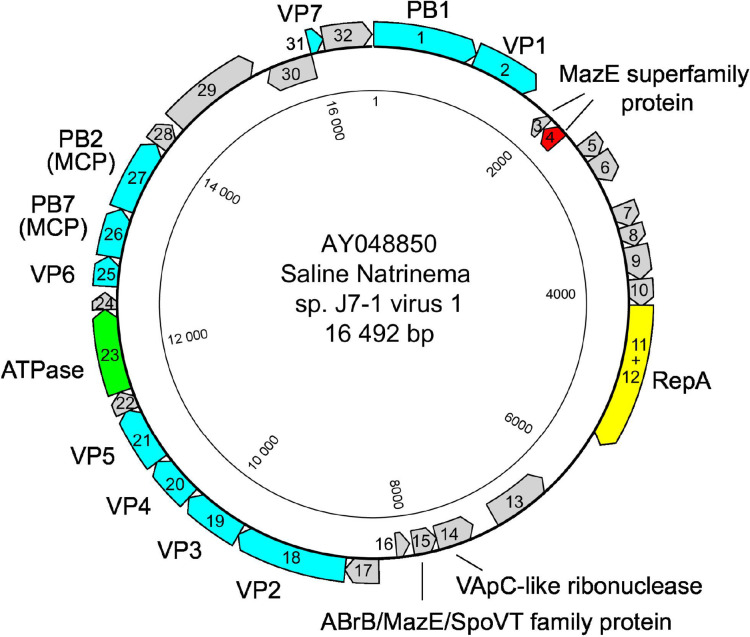
Genome map of Saline Natrinema sp. J7‐1 virus 1. Cyan, structural proteins; yellow, replication initiation protein RepA; green, putative genome packaging ATPase; red, transcriptional regulator controlling virus lysis–lysogeny switch.

The circular dsDNA genome of members of the family *Simuloviridae* is 16–19 kb with about 30 ORFs (Fig. 2). Simuloviruses share 17–21 homologous ORFs including replication, regulation and virion structure-related genes; these gene modules are generally collinear among simuloviruses [3].

## Replication

Members of the family *Simuloviridae* have a temperate life cycle, their proviruses residing in host cells as extrachromosomal episomes [1,3, 4]. The Saline Natrinema sp. J7‐1 virus 1-encoded ORF4 is a transcriptional regulator belonging to the MazE superfamily and controls the lysis–lysogeny switch [3]. The virus is triggered to undergo the lytic life cycle upon treatment with the DNA-damaging agent mitomycin C. Homologues of Saline Natrinema sp. J7‐1 virus 1 ORF4 are encoded by Haloterrigena jeotgali icosahedral virus 1 and Natrinema versiforme icosahedral virus 1, implying that simuloviruses employ a common lysis–lysogeny regulatory mechanism [3]. Saline Natrinema sp. J7‐1 virus 1 genome replication uses a rolling-circle mechanism, which depends on the virus-encoded replication initiation protein RepA belonging to the HUH endonuclease superfamily [5,6]. A RepA homologue has been identified in Haloterrigena jeotgali icosahedral virus 1, but not in Natrinema versiforme icosahedral virus 1. Production of Saline Natrinema sp. J7‐1 virus 1 virions causes cell lysis. Simuloviruses infect halophilic archaea of the genera *Natrinema* and *Haloterrigena*, order Natrialbales.

## Taxonomy

Current taxonomy: ictv.global/taxonomy. The family *Simuloviridae* includes a single genus, *Yingchengvirus*, with three species.

## Resources

Full ICTV Report on the family *Simuloviridae*: ictv.global/report/simuloviridae.
